# Nationwide seroprevalence of canine vector-borne pathogens in dogs in Spain

**DOI:** 10.1186/s13071-026-07500-3

**Published:** 2026-06-14

**Authors:** Ana Montoya-Matute, Rocío Checa, Clara Gómez-Velasco, Rosa Gálvez, Isabel Mendoza, Juliana Sarquis, Juan P. Barrera, Efrén Estévez-Sánchez, Daniela García-Rupérez, Guadalupe Miró

**Affiliations:** 1 Animal Health Department, Veterinary Faculty, Universidad Complutense de Madrid, Madrid, Spain; 2 Microbiology and Parasitology Department, Pharmacy Faculty, Universidad Complutense de Madrid, Madrid, Spain; 3 Department of Specific Didactics, School of Education and Teacher Training, Universidad Autónoma de Madrid, Madrid, Spain

**Keywords:** *Leishmania infantum*, *Ehrlichia canis*, Seropositivity, *Dirofilaria immitis*, Positivity, Dog, CVBD (Canine Vector Borne-Diseases), Spain

## Abstract

**Background:**

Canine vector-borne diseases (CVBDs) are a health risk for both dogs and humans. This nationwide study sought to determine the seropositivity of *Leishmania infantum* and *Ehrlichia canis* and the positivity of *Dirofilaria immitis*, the main vector-borne pathogens prevalent in Europe, in apparently healthy dogs from Spain. Possible associations between seropositivity/positivity and epidemiological variables were also assessed, respectively.

**Methods:**

In a survey conducted in all Spanish 50 provinces, 11,886 dogs from 609 veterinary clinics were tested using the URANOvet® diagnostic rapid test to detect *D. immitis* antigen, and antibodies against *L. infantum* and *E. canis*. Data were collected regarding sex, age, habitat, clinical signs compatible with each pathogen and the regular use of ectoparasiticides.

**Results:**

The overall seropositivity was 17.3% (1915/11,048) for *L. infantum,* and 3.4% (315/9,125) for *E. canis.* Positivity for *D. immitis* was 3.2% (314/9,938). A considerable proportion of infected dogs showed no clinical signs, representing 17.7% of *L. infantum*, 64.1% of *D. immitis*, and 35.9% of *E. canis* positive cases. Significant differences in the epidemiological variables examined (*P* < 0.05) were related to seropositivy/positivity for the three pathogens examined, including geographic location, habitat, associated clinical signs and use of ectoparasiticides. While a higher seropositivity of *L. infantum* and positivity for *D. immitis* antigen were recorded in older dogs (*P* < 0.001), male dogs showed a higher seropositivity of *L. infantum* (*P* < 0.001).

**Conclusions:**

These data suggest that dogs in Spain are consistently exposed to all three vector-borne pathogens analysed. Veterinarians should include these CVBDs in their differential diagnoses and encourage the use of repellents and other prophylactic measures to prevent their transmission by arthropod vectors, considering regional epidemiological risk and lifestyle. Our findings also highlight the need for early detection by routine screening of clinically healthy dogs, as they could be important subclinical carriers.

**Graphical Abstract:**

**Figure:**
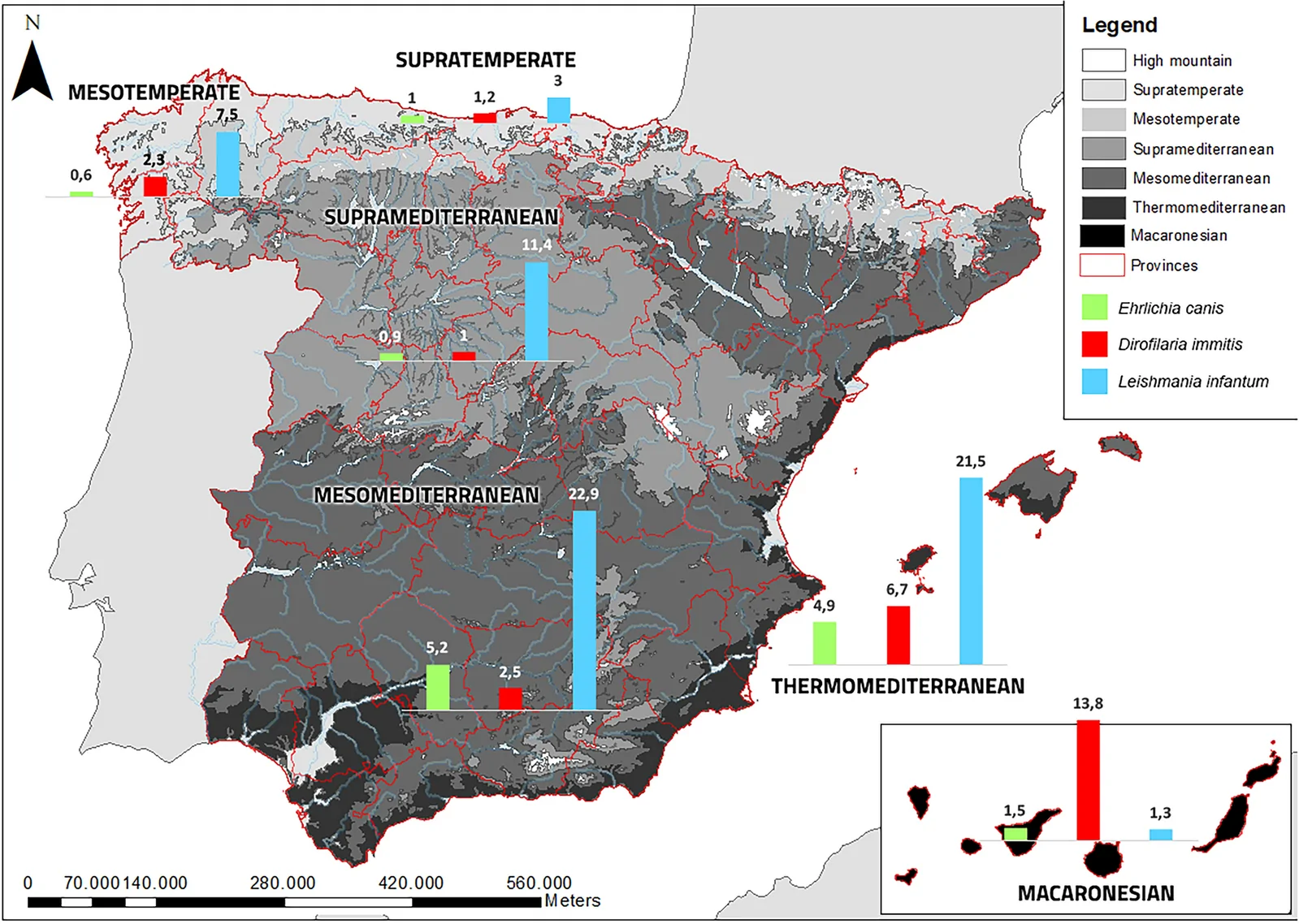

**Supplementary Information:**

The online version contains supplementary material available at 10.1186/s13071-026-07500-3.

## Background

Canine vector-borne diseases (CVBDs) include a wide variety of infectious diseases whose aetiological agents are transmitted by arthropod vectors, such as ticks, fleas, lice and Diptera, including mosquitoes and phlebotomine sandflies. Many of these CVBDs are important, not only because they cause disease in pets but because they may be zoonotic. Among these, dirofilariosis and leishmaniosis, caused by *Dirofilaria* spp. and *Leishmania infantum,* respectively, which are well-recognized zoonotic pathogens in the Mediterranean region [1–4].

The distributions of CVBDs are determined by the presence of a competent vector and a natural host reservoir (including wild or domestic animals). In recent years, vector arthropods have been described in regions previously considered free of these CVBDs and their vectors, mainly due to climate change and ecological factors [5]. Furthermore, regulations regarding the management of stray and wild animals, together with the movement of pets, could also be having a major impact on the epidemiological situation of vector-borne diseases [6–8]. In effect, the expansion of endemic areas has been reported for various parasite diseases, such as dirofilariosis and leishmaniosis [9, 10].

The effective control of CVBDs requires a thorough understanding of the pathogens involved, their vectors and their main hosts, so that preventive measures can be designed. The present study focuses on three of the main CVBDs affecting dogs at our latitudes: leishmaniosis, dirofilariosis and ehrlichiosis.

Canine leishmaniosis (CanL) is a zoonotic disease caused by the protozoan *Leishmania infantum* and transmitted by phlebotomine sandflies in Southern Europe. Dogs are its main peridomestic host. The distribution of *L. infantum* infection in dogs in Spain is partially associated with the climate conditions of its different regions, which can be more or less suitable for the survival of its sand fly vector (temperatures not lower than 17–18 °C) [11]. In Spain, *L. infantum* seroprevalences range from 2% to 3.3% in northern regions, and from 34.6% to 57.1% in southern and southeastern regions [12]. A dog infected with *L. infantum* can show a wide range of clinical signs, such as apathy, weight loss, enlarged lymph nodes, skin lesions (exfoliative dermatitis, ulcerative dermatitis), ocular disorders and chronic kidney disease, among others [13, 14]. Some of these clinical signs are non-specific and resemble those produced by other CVBDs in dogs. Moreover, not all infected dogs will develop the disease and endemic areas may feature high numbers of clinically healthy carriers [15].

Heartworm disease caused by the nematode *Dirofilaria immitis* is transmitted by mosquitoes of the genera *Aedes, Anopheles* and *Culex*, and is endemic in Spain and other southern European countries [16]. As its vectors are not host specific, many mammals—including humans—can become infected, although the parasite cannot reach maturity in humans. The overall prevalence of *D. immitis* infection reported in dogs living in central Spain (Madrid) is 3% [17], but higher prevalences (> 11%) have been cited in the hyperendemic areas of Canary Islands, Balearic Islands or Salamanca [18, 19]. Similarly, in Greece, prevalence variation depending on the geographical region has been described as 0.7–25% [20]. In contrast, a low prevalence was found in apparently healthy dogs from Croatia (0.4%) [21]. While heartworm disease is chronic, high parasite burdens may induce severe acute disease. In endemic areas, many dogs can have subclinical infection for several months, even years. In sick dogs, clinical signs include intermittent coughing, exercise intolerance, dyspnoea, tachypnea and weight loss [22].

*Ehrlichia canis* is the aetiological agent of canine monocytic ehrlichiosis (CME). This gram-negative rickettsia infects monocytes and is transmitted by ticks from the *Rhipicephalus sanguineus* complex. Therefore, disease distribution is linked to the presence of this hard tick and its habitat range. In Europe, the seroprevalence rate for *E. canis* infection in dogs ranges from 0.2% in Hungary [23] and Russia [24], to 46% and 50% in shelter dogs from Italy [25] and Portugal [26], respectively. In Spain, an overall seropositivity rate of 4–5% has been described [27–29], but higher seroprevalences have been reported in dogs from northeastern Spain [30] (16.7%) and in shelter dogs from Orense in North West Spain (54.7%) [31]. CME shows a severity varying from subclinical infection to chronic disease. Its wide variety of clinical signs includes pale mucous membranes, fever, splenomegaly, asthenia, weight loss, diarrhoea, haematuria, epistaxis, anaemia and thrombocytopenia [32].

This nationwide study aimed to evaluate, in owned dogs across all 50 Spanish provinces, the seropositivity to leishmaniosis (*Leishmania infantum,* Li), and ehrlichiosis (*Ehrlichia canis*, Ec) in order to assess exposure to both pathogens, as well as the positivity for heartworm (*Dirofilaria immitis*, Di) infection to evaluate the potential damage caused by adult worms in their final location, the pulmonary arteries. Different epidemiological variables related to these prevalences were also assessed.

## Methods

### Study design

Dogs included in this study were patients of 609 veterinary clinics randomly selected across the whole country. This was done to ensure that the sample was proportional to the size of the dog population in each province. The sample collection period was from January 2018 to December 2022. From these veterinary practices, 11,886 dogs were selected with the help of the veterinarians responsible for each animal. Inclusion criteria were dogs of any breed, sex and age, healthy dogs and/or dogs showing clinical signs suggestive of CVBD. According to the clinical experience of the investigators (veterinarians), the specific clinical signs for the 3 pathogens were limited as follows: for *L. infantum*: asthenia, cutaneous lesions and lymphadenomegaly; for *D. immitis*: asthenia, exercise intolerance and coughing; for *E. canis*: asthenia, pale mucous membranes and fever. Dogs presenting these clinical signs were classified as having 'compatible clinical signs' for the respective pathogen. Dogs under 6 months of age were excluded, because there may not have been sufficient time to detect infection due to the prepatent periods of *D. immitis* and *L. infantum*, and because there have been few reported cases of vertical transmission for *L. infantum*.

A clinical data sheet was designed to collect information for each dog regarding: sex, age, breed, habitat, travel history, clinical signs, presence of ectoparasites (e.g., fleas, ticks) and use of ectoparasiticides.

Preventive measures against vector-borne diseases were recorded when available. These included the use of topical or collar-based ectoparasiticides with proven efficacy against sandflies (*L. infantum*), ticks (*E. canis*), and mosquitoes (*D. immitis*), as well as macrocyclic lactones (e.g., milbemycin oxime, moxidectin, ivermectin) with microfilaricidal activity for heartworm prevention and oral isoxazolines as systemic acaricides. Information on prophylactic use was self-reported by owners, so the specific type of product, frequency of administration, duration of protection or adherence over time could not be verified. This meant that this variable was treated as descriptive and exploratory rather than as a reliable indicator of effective prophylaxis.

Habitat was classified according to the owner's report of where the dog spent most of its time. 'Indoor' dogs were defined as those living primarily inside the home with only limited outdoor access (e.g., for walks), 'outdoor' dogs as those living primarily outside the home (e.g., in gardens, kennels or rural properties) for most of the day and night, and 'both' as dogs that regularly spent significant time indoors and outdoors (e.g., indoor dogs with free access to a terrace).

The study protocol complied with international regulations for biomedical research on animals. Accordingly, signed informed consent was required from the owners and responsible veterinarians before clinical examination and blood collection in each dog.

The number of veterinary clinics per province ranged from 1 to 60 (median: 14.06 clinics), and the number of dogs tested per province ranged from 15 to 1,265 (median: 237.5 dogs). More detailed information is provided in Supplementary Table S1. Veterinary clinics were randomly selected across the country, targeting broad geographic representation. Within each clinic, veterinarians were instructed to include dogs consecutively seen according to the inclusion criteria.

### Study area

The study was carried out in randomly selected veterinary clinics in all Spanish provinces (*n* = 50), including mainland Spain and the Balearic and Canary Islands.

Spain has a wide range of bioclimatic conditions with nine traditionally defined bioclimatic zones in the Iberian Peninsula and Balearic and Canary Islands [33]. The mesomediterranean, thermomediterranean and supramediterranean zones feature milder temperatures. The mesotemperate and supratemperate zones of the Eurosiberian region provide a transitional climate, while the macaronesian bioclimate of the Canary Islands adds a subtropical component. The bioclimatic classification used in this study is based on data from the Spanish Ministry for Ecological Transition and Demographic Change and the scheme of Rivas-Martínez et al. [33]. Distributions of the vector-borne diseases examined were assessed according to this climate and geographical diversity.

### Sample and data collection

Each dog was thoroughly examined by the practitioner to determine its health status and eligibility for inclusion in this study. As no standardized scoring system was employed across all participating clinics, the classification of dogs as having mild, moderate or severe clinical signs was based on the attending veterinarian's clinical judgement and should, therefore, be interpreted as subjective. Results of the clinical exam and anamnesis were recorded in the clinical data collection sheet (Supplementary S2).

From each dog, approximately 1–3 ml of blood were collected from the cephalic or jugular vein and processed immediately for performing rapid in-house tests.

### Serological testing

Blood sampling and further serological tests were performed by the veterinarians using individual rapid immunochromatography tests (URANOvet, Barcelona, Spain) in blood (EDTA-anticoagulated), serum or plasma samples. All participating veterinarians followed the manufacturer's standardised instructions regarding sample volume, incubation time and interpretation of results.

Uranotest *Dirofilaria*® (sensitivity 94.4%; specificity 100% as reported the manufacturer, although independent studies suggest that cross-reactivity with other nematode may occur) [34] to detect *D. immitis* antigens, Uranotest *Leishmania*® (sensitivity 96.7%; specificity 98.8%) [35] to detect antibodies against *L. infantum*; and Uranotest *Ehrlichia*® (sensitivity 95%; specificity 94.6%) to detect antibodies against *E. canis*. Sensitivity and specificity values were provided by the manufacturer (www.uranobiotech.com).

Test results were recorded in the clinical examination form.

### Statistical and spatial analysis

All statistical tests were conducted using the package IBM SPSS Statistics version 25.0 (IBM Corporation, New York, USA). Descriptive analyses were performed using standard statistical methods for qualitative variables (absolute and relative frequencies) and quantitative variables (mean and standard deviation).

Multivariable logistic regression models were constructed for each pathogen to calculate adjusted odds ratios (aORs). The models included the following variables: age; sex; neuter status; habitat; clinical signs compatible with each CVBD; presence of ectoparasites; use of ectoparasiticides; and province. Variables were selected using the enter method and model fit was assessed using omnibus tests, Cox & Snell R^2^ and Nagelkerke R^2^.

Additionally, seropositivity rates were compared using chi-squared tests. Figure 4 prepares using the Spatial Analyst (SAA) application in ArcGis v.10.7.1 GIS (ESRI, Redlands, CA). Each Spanish province was assigned to the bioclimate described for the largest area within the province. Significance was set at *P* < 0.05.

## Results

### Signalment of dogs

Out of 11,886 dogs enrolled, 5,635 (47.6%) were female (1,649 neutered and 3,986 intact) and 6,200 (52.2%) were male (1,442 neutered and 4758 intact). Age ranged between 6 months and 18 years (mean age: 5.7 years) with four age groups established: < 1 year (10.9%; 1,283/11,719), 1–3 years (22.8%; 2,673/11,719), 3–7 years (35.4%; 4,150/11,719) and > 7 years (30.8%; 3,613/11,719).

According to the epidemiological data collected: 43.7% (5,164/11,829) lived indoors, 40.8% (4,853/11,829) outdoors and 15.2% (1,812/11,829) both (outdoors and indoors). Additionally, 13.7% (1,605/11,748) had an ectoparasite infestation, of which 45.1% (701/1,554) were infested by ticks, 42.6% (662/1,554) by fleas, and 12.3% (191/1,554) by fleas and ticks. 74.3% (8,423/11, 342) of the dogs had been treated with different anti-ectoparasite drugs with acaricidal, repellent and/or insecticidal effects. Among these treated dogs, 81.9% (6,310/7,702) were protected against ticks, fleas, mosquitoes, and sandflies, 17.1% (1,316/7,702) against ticks and fleas, 0.9% (67/7,702) against fleas and 0.1% (9/7,702) against ticks.

#### *Leishmania infantum *seropositivity

**Fig. 1:**
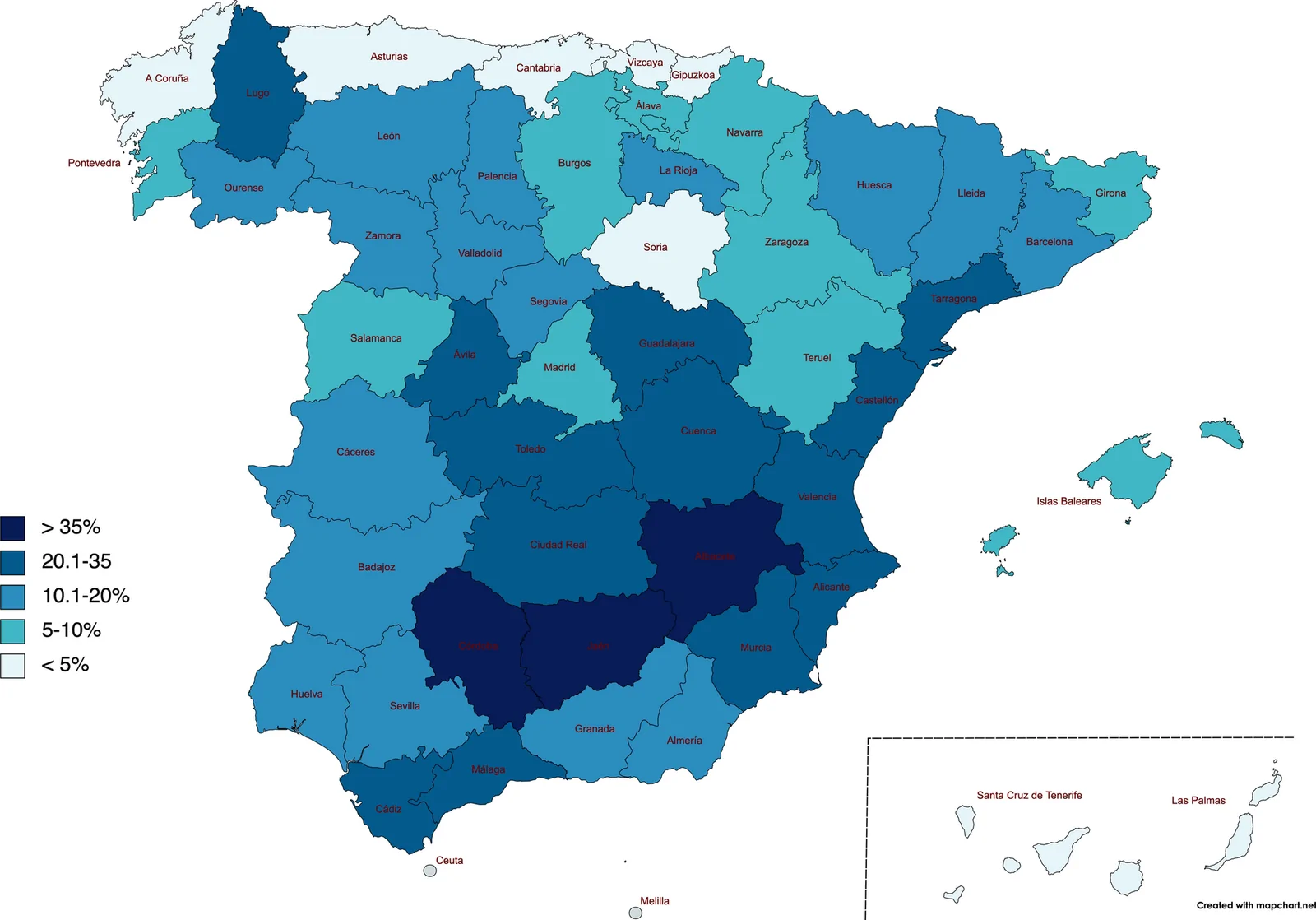
*Leishmania infantum* seropositivity results for each Spanish province

Overall seropositivity for *L. infantum* was 17.3% (1,915/11,048). Seroprevalence data by province and bioclimatic zones are provided in Figs. 1 and 4, respectively. Significant differences were observed among Spanish provinces (*P* < 0.001). The highest seroprevalence rates were detected in the southeastern provinces of Córdoba (58.2%; 53/91), Jaen (43.1%; 44/104) and Albacete (40.6%; 69/170), while lowest seroprevalences were found in the Canary Islands (1.3%; 5/387) and the northern provinces of Cantabria (2.2%; 3/136) and A Coruña (2.4%; 4/167) (Fig. 1). According to bioclimatic zones (Fig. 4), highest seroprevalences were detected in the mesomediterranean (22.9%; 1,354/5,905) and thermomediterranean bioclimates (21.5%; 190/883), followed by medium seroprevalences in the supramediterranean (11.4%; 319/2,789) and mesotemperate (7.5%; 30/402) bioclimates, while lowest seroprevalences were detected in supratemperate (3%; 17/572) and macaronesian bioclimates (1.3%; 5/387) (Table 1).

**Table 1:** Seropositivity of *Leishmania infantum* and *Ehrlichia canis* antibodies and positivity of *Dirofilaria immitis* antigen in dogs according to bioclimate zones

*Bioclimate area*	% [95% CI] (Positive/Total)		
	*L. infantum*	*D. immitis*	*E. canis*
*Supratemperate*	3 [1.8–4.8] (17/572)	1.2 [0.5–2.5] (7/584)	1 [0.4–2.0] (8/796)
*Mesotemperate*	7.5 [5.1–10.5] (30/402)	2.3 [1.1–4.3] (9/400)	0.6 [0.1–2.1] (2/352)
*Supramediterranean*	11.4 [10.3–12.7] (319/2789)	1 [0.7–1.5] (23/2263)	0.9 [0.6–1.4] (20/2136)
*Mesomediterranean*	22.9 [21.8–24.0] (1354/5905)*	2.5 [2.1–2.9] (127/5120)	5.2 [4.6–5.9] (242/4639)*
*Thermomediterranean*	21.5 [18.8–24.4] (190/883)*	6.7 [5.1–8.7] (51/763)*	4.9 [3.4–6.8] (35/719)*
*Macaronesian*	1.3 [0.4–3.0] (5/387)	13.8 [11.3–16.6] (97/702)*	1.5 [0.5–3.2] (6/408)
*Statistical analysis*	*χ*^*2*^ = 561.17, *df* = 5, *P* < 0.001	*χ*^*2*^ = 371.25, *df* = 5, *P* < 0.001	*χ*^*2*^ = 137.63, *df* = 5, *P* < 0.001

CI: confidence interval; *χ*^2^ chi-square value, *df* degrees of freedom. Statistical analysis by Pearson's chi-square test; significance level *P* < 0.05.*

Of the 1,915 dogs testing seropositive for *L. infantum*, 336 (17.5%) showed no clinical signs (*P* < 0.001) (Table 2). While, of the 7,840 healthy dogs, 332 (4.2%) tested positive. This suggests that a substantial proportion of these dogs could act as clinically healthy carriers of the infection.

**Table 2:** Seropositivity of *Leishmania infantum* and *Ehrlichia canis* antibodies and positivity of *Dirofilaria immitis* antigen in dogs and related clinical signs

	Clinical signs	% [95% CI] (*n*)		Statistical analysis
		Positive	Negative	
*L. infantum*	Absent	17.5 [15.9–19.3] (336/1,915)	82.2 [81.4–83.0] (7,512/9,133)	*χ*^*2*^ = *1,245.3, df* = *1, P* < *0.001*
	Present	82.5 [80.7–84.1] (1,579/1,915)	17.8 [17.0–18.6] (1,621/9,133)	
*D. immitis*	Absent	64.0 [58.4–69.3] (201/314)	97.5 [97.2–97.8] (9,398/9,637)	*χ*^*2*^ = *1,487.6, df* = *1, P* < *0.001*
	Present	36.0 [30.7–41.6] (113/314)	2.5 [2.2–2.8] (239/9,637)	
*E. canis*	Absent	35.9 [30.5–41.6] (113/315)	89.2 [88.5–89.8] (7,889/8,848)	*χ*^*2*^ = *987.4, df* = *1, P* < *0.001*
	Present	64.1 [58.4–69.5] (202/315)	10.8 [10.2–11.5] (959/8,848)	

CI: confidence interval; *χ*^2^ Chi-square, *df* degrees of freedom. Statistical analysis based on Pearson's chi-square test; significance level *P* < 0.05

Some significant associations were detected with the epidemiological variables considered (Table 3). Accordingly, the seroprevalence of *L. infantum* infection was significantly lower (*P* < 0.001) in dogs under 1 year of age (7.3%; 87/1,196), while in the other age groups, prevalences ranged from 16.1% to 21.4%. Significant differences were also detected according to sex (*P* < 0.001), with a higher seroprevalence in males (18.8%) compared to females (15.8%). In addition, intact dogs (19.4%) showed a significantly higher percentage of seropositivity compared to neutered dogs (11.7%) (*P* < 0.001).

**Table 3:** Seropositivity of *Leishmania infantum* antibodies according to epidemiological variables

Epidemiological variable		% [95% CI] (Positive/Total)	Adjusted OR (95% CI)	Statistical analysis
Age (years)	< 1	7.3 [5.9–8.9] (87/1196)	Ref	*χ*^*2*^ = *132.03, df* = *3, P* < *0.001*
	1–3	17.8 [16.3–19.4] (439/2472)	2.717 (1.997–3694)	
	3–7	21.4 [20.1–22.7] (824/3856)	3.179 (2.366–4.272)	
	> 7	16.1 [15.9–18.4] (543/3375)	2.224 (1.647–3.005)	
Sex	Female	15.8 [14.8–16.8] (829/5250)	Ref	*χ*^*2*^ = *17.01, df* = *1, P* = *0.032*
	Male	18.8 [17.8–19.8] (1083/5764)	1.150 (1.012–1.306)	
Neutered	Yes	11.7 [10.5–13.0] (336/2880)	Ref	*χ*^*2*^ = *87.77, df* = *1, P* < *0.001*
	No	19.4 [18.5–20.3] (1575/8124)	1.385 (1.184–1.621)	
Habitat	Indoor	9.6 [8.8–10.5] (468/4857)	Ref	*χ*^*2*^ = *486.22, df* = *2, P* < *0.001*
	Outdoor/Indoor	14.6 [12.9–16.4] (246/1680)	1.288 (1.025–1.618)	
	Outdoor	26.8 [25.5–28.1] (1194/4460)	2.349 (1.972–2.799)	
Preventive treatment	Yes	13.8 [12.9–14.7] (829/6028)	Ref	*χ*^*2*^ = *118.17, df* = *1, P* < *0.003*
	No	21.6 [20.5–22.8] (1086/5020)	1.726 (1.327–2.245)	

OR: odds ratio; CI: confidence interval; Ref: reference category; *χ*^2^: Chi-square value, *df*: degrees of freedom. Univariate associations were assessed by Pearson’s χ^2^ test; odds ratios and 95% confidence intervals were derived from a multivariate logistic regression model. Significance level *P* < 0.05

Environmental and preventive factors also play a key role. Hence, seroprevalence was significantly higher in dogs living outdoors (26.8%) (*P* < 0.001) than indoors (9.6%) or both in- and outdoors (14.6%), with a 2.3-fold higher risk in outdoor dogs [*P* < 0.001; OR: 2.3 (95% CI 1.9–2.8)]. Furthermore, the proportion of seropositive dogs that were not applied sandfly repellents (e.g., collar or *spot-on*) was significantly higher (21.6%) than those subjected to regular preventive measures (13.8%) [*P* < 0.001; OR: 1.73 (95% CI 1.56–1.92)].

#### *Dirofilaria immitis* infection

**Fig. 2:**
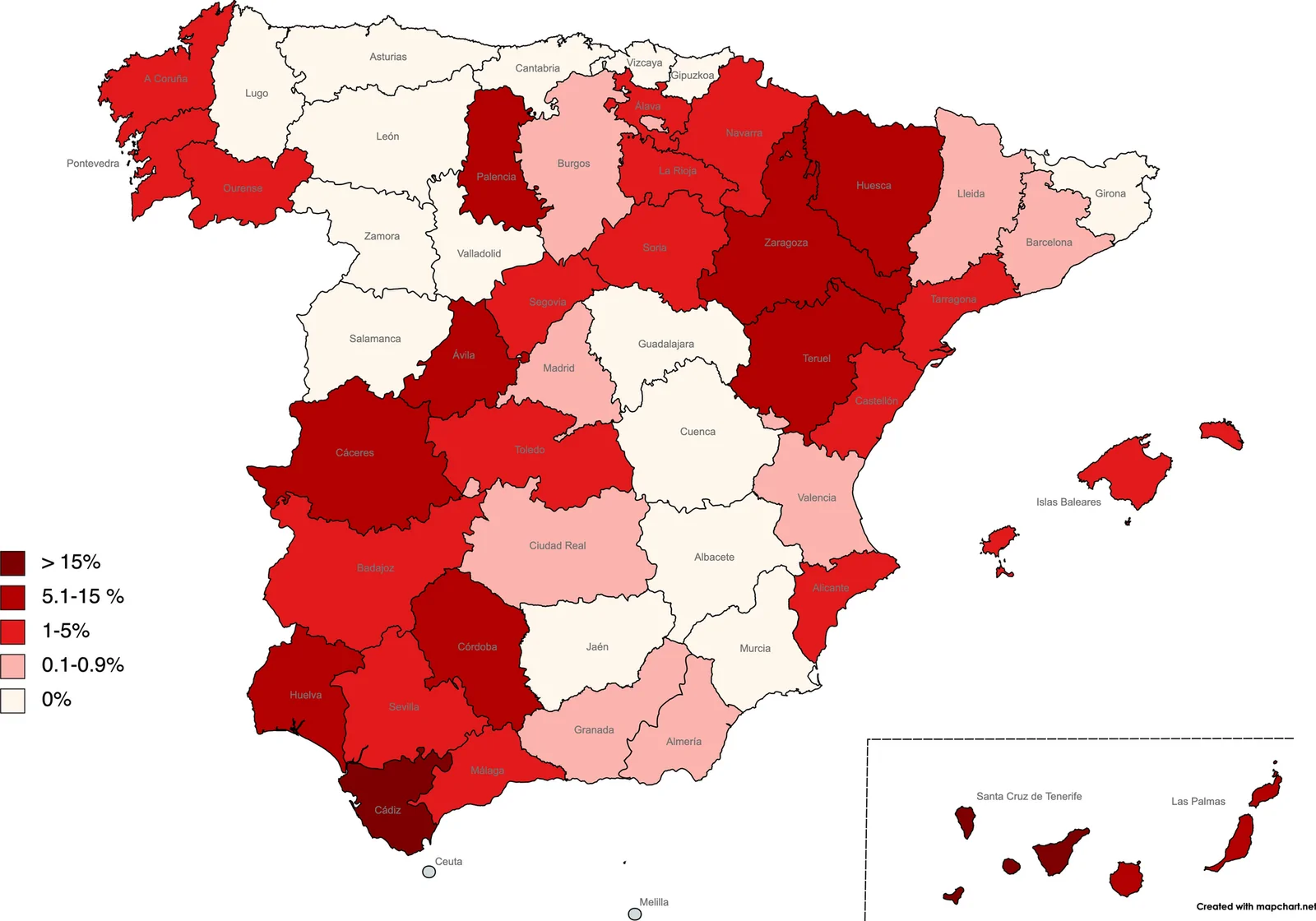
*Dirofilaria immitis* positivity results for each Spanish province

Overall positivity for *D. immitis* antigen was 3.2% (314/9,951). Prevalence data by province are provided in Fig. 2 (*P* < 0.001); the highest prevalences were noted in the Andalusian province of Cádiz (24.4%; 31/127) and in the Canary Islands, particularly Tenerife (18.6%; 72/387) and Las Palmas (13.3%; 22/165). It is important to emphasize that in 20 Spanish provinces, dogs were tested, but all were negative. By bioclimatic zone (Fig. 4), highest prevalences were detected in the Macaronesian bioclimate (13.8%; 97/702), followed by intermediate seroprevalences in the thermomediterranean bioclimate zone (6.7%; 51/763), and finally, lower seroprevalences in mesomediterranean (2.5%, 127/5120), mesotemperate (2.3%, 9/400) and supratemperate zones (1.2%, 7/584) (Table 1).

Of the 314 positive dogs, more than half of these dogs testing positive for *D. immitis* showed no clinical signs (64%; 201/314), while 8.9% (28/314) exhibited severe clinical signs (*P* < 0.001) (Table 2).

When we considered epidemiological factors, age was found to be significant (*P* = 0.018), with a lower prevalence observed in dogs under 1 year of age (1.5%) compared to older age groups (3–3.4%). No significant differences were observed by sex (*P* = 0.831). Intact dogs (3.7%) showed a higher prevalence compared to neutered ones (1.7%) (*P* < 0.001) (Table 4).

**Table 4:** Positivity of *Dirofilaria immitis* antigen detection according to epidemiological variables

Epidemiological variable		% [95% CI] (Positive/Total)	Adjusted OR (95% CI)	Statistical analysis
*Age (years)*	< 1	1.5 [0.9–2.5] (16/1050)	Ref	*χ*^*2*^ = 10.08, *df* = 3, *P* = 0.018
	1–3	3.4 [2.7–4.3] (77/2244)	2.054 (1.033–4.087)	
	3–7	3.3 [2.7–3.9] (114/3482)	1.721 (0.880–3.363)	
	> 7	3 [2.4–3.6] (91/3063)	1.800 (0.911–3.556)	
*Sex*	Female	3.1 [2.6–3.7] (147/4714)	NS	*χ*^*2*^ = 0.02, *df* = 1, *P* = 0.876
	Male	3.2 [2.7–3.7] (166/5198)		
*Neutered*	Yes	1.7 [1.3–2.3] (45/2640)	NS	*χ*^*2*^ = 24.21, *df* = 1, *P* < 0.001
	No	3.7 [3.3–4.1] (268/7272)		
*Habitat*	Indoor	1.3 [1.0–1.7] (55/4245)	Ref	*χ*^*2*^ = 94.56, *df* = 2, *P* < 0.001
	Outdoor/Indoor	3.4 [2.6–4.5] (53/1556)	2.749 (1.702–4.442)	
	Outdoor	5 [4.4–5.7] (206/4105)	2.973 (1.714–5.157)	
*Preventive treatment*	Yes	2.2 [1.8–2.6] (116/5391)	NS	*χ*^*2*^ = 38.07, *df* = 1, *P* < 0.001
	No	4.3 [3.8–4.9] (198/4560)		

OR: odds ratio; CI: confidence interval; Ref: reference category; *χ*^2^ chi-square value, *df* degrees of freedom. NS: not significant in multivariable model. Univariate associations were assessed by Pearson’s χ^2^ test. Odds ratios and 95% confidence intervals were derived from a multivariate logistic regression model. Significance level *P* < 0.05

When analysing *D. immitis* prevalences by habitat, only 1.3% indoor dogs tested positive, while this percentage was higher in dogs living outdoors (5%) or both in- and outdoors (3.4%) (*P* < 0.001); the risk of being infected by *D. immitis* was three times greater in outdoor dogs (OR: 2.97; CI 95%, 1.7–5.2). Dogs without prophylactic measures (microfilaricides macrocyclic lactones and insect repellents) showed a higher prevalence (4.3%), compared to dogs subjected to these preventive measures (2.2%) (*P* < 0.001). (Table 4).

#### *Ehrlichia canis* seropositivity

**Fig. 3:**
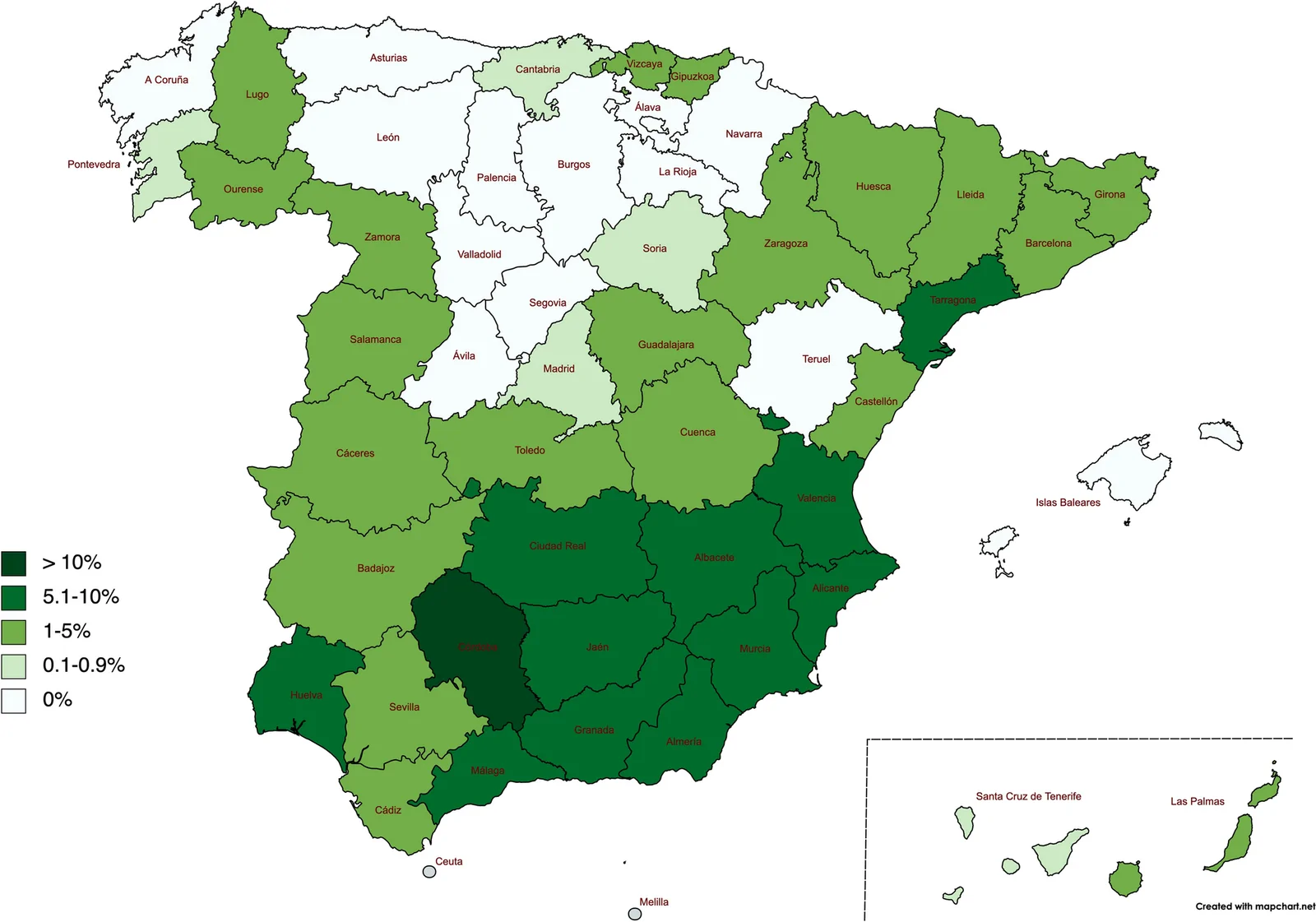
*Ehrlichia canis* seropositivity results for each Spanish province

**Fig. 4:**
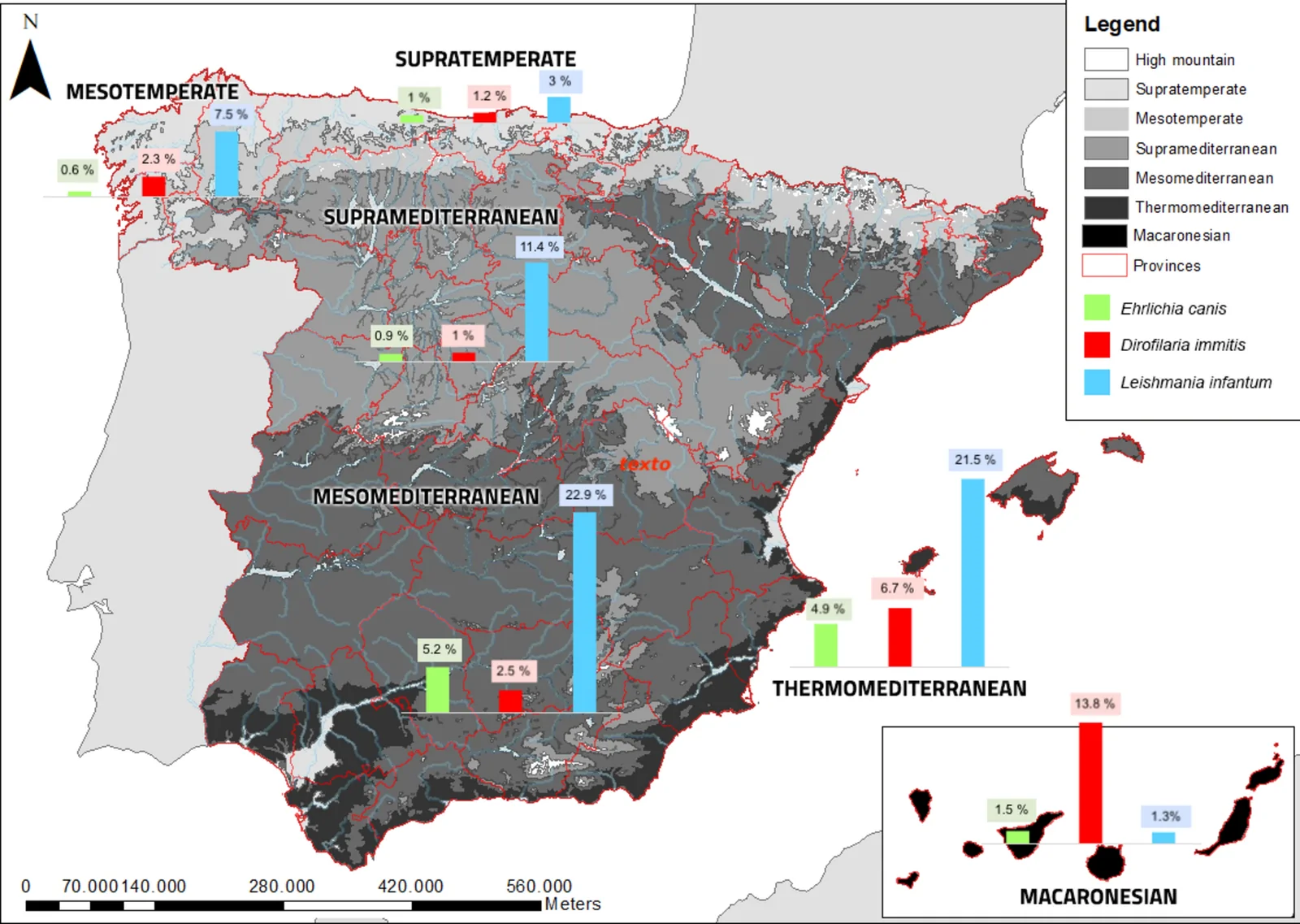
Seropositivity of *Leishmania infantum* and *Ehrlichia canis antibodies *and positivity of *Dirofilaria immitis *antigen in dogsaccording to bioclimate zones

Overall seropositivity for *E. canis* was 3.4% (315/9,163), ranging from 0% to 11.3% depending on the province (Fig. 3) (*P* < 0.001). Highest seroprevalences were found in the southwestern provinces of Córdoba (11.3%; 7/62), Huelva (11%; 11/100) and Albacete (10%; 12/120), while no seropositive data were recorded in 16 provinces (Fig. 3). In terms of bioclimate zones (Fig. 4), intermediate seroprevalences were detected in the meso- (5.2%; 242/4,639) and thermomediterranean bioclimates (4.9%; 35/719), and lower seroprevalences were detected in the mesotemperate (0.6%; 2/352), supramediterranean (0.9%; 20/2,136), supratemperate (1%; 8/796), and macaronesian zones (1.5%; 6/408) (Table 1).

Of 9,163 dogs examined, 1,161 presented clinical signs associated with *E. canis* infection (59.2% mild, 28.2% moderate, 12.6% severe). However, of 315 seropositive dogs for *E. canis*, 35.9% (113/315) were apparently healthy (Table 2).

No significant differences were found between *E. canis* seroprevalence and age (*P* = 0.150) or sex (*P* = 0.171); however, a higher seroprevalence was observed in non-neutered dogs (3.7%) compared to neutered dogs (2.7%) (*P* = 0.025). Significant differences were also found between *E. canis* seroprevalence and habitat (*P* < 0.001), the seroprevalence being higher in dogs living outdoors (4.7%) compared to indoors (2.5%) or with mixed access (2.6%), with a 1.7 times higher risk in outdoor dogs (OR: 1.67; CI 95%: 1.49–2.8) (Table 5).

**Table 5:** Seropositivity of *Ehrlichia canis* antibodies according to epidemiological variables

Epidemiological variable		% [95% CI] (Positive/Total)	OR (95% CI)	Statistical analysis
*Age (years)*	< 1	2.3 [1.5–3.4] (22/976)	NS	*χ*^*2*^ = 5.41, *df* = 3, *P* = 0.150
	1–3	3.9 [3.1–4.8] (79/2045)		
	3–7	3.5 [2.9–4.2] (112/3204)		
	> 7	3.6 [2.9–4.3] (102/2873)		
*Sex*	Female	3.2 [2.7–3.7] (138/4371)	NS	*χ*^*2*^ = 1.88, *df* = 1, *P* = 0.171
	Male	3.7 [3.2–4.2] (175/4757)		
*Neutered*	Yes	2.7 [2.1–3.4] (70/2552)	NS	*χ*^*2*^ = 4.75, *df* = 1, *P* = 0.025
	No	3.7 [3.3–4.2] (243/6576)		
*Habitat*	Indoor	2.5 [2.0–3.0] (97/3877)	Ref	*χ*^*2*^ = 31.56, *df* = 2, *P* < 0.001
	Outdoor/Indoor	2.6 [1.9–3.6] (37/1415)	1.088 (0.693–1.709)	
	Outdoor	4.7 [4.1–5.4] (181/3850)	1.673 (1.226–2.283)	
*Tick infestation history*	Yes	10.5 [8.5–12.8] (83/790)	2.044 (1.489–2.804)	*χ*^*2*^ = 127.81, *df* = 1, *P* < 0.001
	No	2.8 [2.4–3.2] (232/8373)	Ref	
*Preventive treatment*	Yes	2.7 [2.3–3.1] (156/5855)	NS	*χ*^*2*^ = 28.58, *df* = 1, *P* < 0.001
	No	4.8 [4.1–5.6] (159/3308)		

OR: odds ratio; CI: confidence interval; Ref: reference category; *χ*^2^ chi-square value, *df* degrees of freedom. NS: not significant in multivariable model. Univariate associations were assessed by Pearson’s χ^2^ test. Odds ratios and 95% confidence intervals were derived from a multivariate logistic regression model. Significance level *P* < 0.05

The link between the presence or absence of ticks on dogs and *E. canis* infection was also analyzed revealing a higher seropositivity in dogs infested with ticks (10.5%) compared to those without ticks (2.8%) (*P* < 0.001). Similarly, the seroprevalence was higher in dogs with no tick prevention measures (4.8%) versus those subjected to these measures (2.7%) (*P* < 0.001) (Table 5). Unfortunately, ticks were not identified in the present study due to economic restrictions.

#### Co-infections

A significant association was observed between *L. infantum* and *E. canis* seropositivity, affecting 118 dogs (1.3% of the total). Of these, 104 (88.1%) had compatible clinical signs, this proportion was significant higher compared to both *L. infantum* (82.5%; *P* = 0.041) and *E. canis* individually (64.1%; *P* < 0.001). Regarding *L. infantum–D. immitis* co-positivity (44 dogs; 0.4%), 36 (81.8%) had clinical signs and 8 (18.2%) did not; representing a significantly higher rate of clinical signs than for *D. immitis* positivity (*P* = 0.03). However, no significant associations were found for *D. immitis–E. canis* co-positivity (8 dogs; 0.08%), where six dogs had compatible clinical signs and two did not (*P* > 0.05). Additionally, six dogs (0.05%) were positive for all three pathogens, of which four had compatible clinical signs and two did not (*P* > 0.05).

## Discussion

This study provides a comprehensive analysis of the seropositivity of *Leishmania infantum* and *Ehrlichia canis* and positivity for *Dirofilaria immitis* antigen, in a large canine population across Spain, highlighting key epidemiological and preventive factors associated with these CVBDs. Although our sampling strategy aimed to ensure geographic areas were represented proportionally to the size of the dog population, some variability in clinic participation and sample sizes occurred due to recruitment constraints across Spain’s diverse geographic and demographic landscape. Our findings confirm their widespread presence and underscore the importance of early diagnostics in healthy dogs, given the high proportion of seropositive animals with subclinical infection.

To our knowledge, this is the first study to determine the prevalence of vector-borne pathogens in dogs across every Spanish province. Previous studies have involved smaller sample sizes and fewer provinces [27–30, 36]. This study offers a broader, more comprehensive perspective, examining 11,886 dogs across all 50 Spanish provinces. In a recent study of the 17 autonomous communities of Spain, Montoya-Alonso et al. [29] found a high seroprevalence of *L. infantum* (10.36%) and *D. immitis* (6.25%) in 4,643 dogs. While these authors highlighted the increasing prevalence of these diseases, particularly in northern regions, by including a large number of veterinary practices (*n* = 609), this study expands these data, covering every province, to provide a more detailed view of regional variation. However, results are difficult to directly compare because of differences in the diagnostic techniques used, the sizes and origins of samples, and the time intervals between them.

The prevalence detected were 17.3% for *L. infantum,* followed by 3.5% for *E. canis* and 3.2% for *D. immitis* antigen, though these rates varied geographically [28, 29]. It is important to clarify the interpretation of these results. Seropositivity for *L. infantum* and *E. canis* indicates exposure or infection but does not necessarily imply clinical disease, as antibodies may persist for months or years after exposure or infection without detecting any clinical sign [32, 37]. For *D. immitis,* a positive antigen test is always indicative of infection, even antigen can persist for months after worm death [38, 39]. Moreover, potential antigen cross-reactivity with other canine nematodes (e.g., *Angiostrongylus vasorum*) cannot be excluded, as parasitological examinations were not routinely performed, which should be considered when interpreting positive results [34].

Several limitations related to the diagnostic approach must be acknowledged. A notable constraint is the use of rapid immunochromatographic tests (URANOVet®), chosen for practicality in this large-scale field study. These tests have recognized limitations, including potential cross reaction (e.g., *Leishmania* species antibodies, *Ehrlichia canis* tests with *Rickettsia* spp. or *Anaplasma* species, and *Dirofilaria immitis* antigen tests with other nematodes) and possibly reduced sensitivity in clinically healthy or subclinically infected dogs, which may lead to an underestimation of true prevalence [34, 69]. We did not perform confirmatory testing (e.g., PCR, Western Blot) or systematic fecal exams to rule out other intestinal infections. Therefore, our reported prevalences, while highly indicative of exposure or infection, should be interpreted with this diagnostic limitation in mind.

Furthermore, the clinical sign classification relied on veterinarians’ assessments rather than standardized criteria, which may affect comparisons between studies. The stratification of clinical signs into mild, moderate, and severe was performed to preliminarily assess whether clinical picture correlated with seropositivity. We acknowledge that dogs were not systematically screened for comorbidities; therefore, recorded clinical signs cannot be attributed solely to the studied CVBDs, representing an important limitation in interpreting disease-specific severity. Although our sampling strategy aimed for proportional geographic representation, variability in clinic participation occurred. We cannot exclude the possibility that diagnostic tests may have been more frequently performed in suspected cases, which would introduce a selection bias. Nevertheless, we confide that the large sample size and wide distribution of veterinary practices across the country will reduce the impact of this limitation on its overall results.

### *Leishmania infantum* seropositivity

Numerous studies have mapped the seroprevalence of canine leishmaniosis in Spain [12]. The present survey includes some previously non-sampled provinces in northern and central Spanish areas (e.g., Cuenca, Huesca, and Teruel) for a more comprehensive understanding of seroprevalence distributions. Overall, the *L. infantum* seropositivity rate detected in this study of 17.3% was notably higher than previous nationwide reports (10.4–10.7%) [29, 56], suggesting possible regional variations, differences in diagnostic methodologies, or increased exposure in certain areas. This was particularly evident in southeastern provinces and the meso- and thermomediterranean bioclimatic zones, in line with previous reports [40, 41]. The upward trend in previously non-endemic areas [14, 42–44] may be linked to climate change, which contributes to creating optimal conditions for the survival of *Phlebotomus perniciosus* in new regions [45–47], raising concerns about the potential autochthonous transmission of vector-borne pathogens in these areas [48–50]. In addition, the presence of the parasite in other animal hosts, such as domestic cats and wild mammals like hares, indicates the potential involvement of multiple animal species in the parasite’s transmission [51].

CanL is a chronic systemic disease whose clinical manifestations vary from subclinical to severe disease. It should be noted that the individual immune response of the dog and virulence of the *L. infantum* isolate are important contributing factors [52]. Consequently, a positive serological test indicates exposure and a humoral response but does not distinguish between clinically healthy infected dogs (which may control the infection via cellular immunity) and those with active disease. In this study, 29% of dogs showed clinical signs, while 17.5% clinically healthy but seropositive, confirming the role of subclinical carriers as potential reservoirs [53]. Many of these clinically healthy seropositive dogs likely developed an effective cellular immune response, allowing them to control the infection without overt clinical disease, as previously described [52–54]. The relevance of clinically healthy infected dogs is supported by the results of recent studies by Baxarias et al. and Müller et al., who reported a significant seroprevalence in apparently healthy dogs [54, 55]. These findings reinforce the importance of regular serological screening, preventive prophylaxis, and adherence to LeishVet recommendations. The Europe-wide study by Miró et al. supports these recommendations, showing that increased screening and preventive measures have contributed to a decline in seropositivity rates for *Leishmania* spp. across Europe [56].

In the present study, differences were detected in *L. infantum* seropositivity according to animal age, such that rates were significantly higher in middle-aged dogs (3–7 years), in agreement with previous reports [55, 57]. However, other authors argue that *L. infantum* infection in dogs shows a bimodal distribution pattern with two peaks of higher seroprevalences (in dogs under 3 years and older than 8 years). The first peak in young dogs (< 3 years) could be related to an immature immune system, making them more vulnerable to infection, while the second peak in older dogs (> 8 years) may result from prolonged exposure, as reported in several studies in the Mediterranean region [15, 58]. No significant impact of sex on seroprevalence has been reported [12, 57]. However, we observed a higher seropositivity rate in males and non-neutered dogs. This could be explained by the association between gender and owner preferences for different dog uses (e.g., males are more often chosen as guard dogs), and these are more likely to be outdoors for longer periods [57]. Other studies have found a higher prevalence of *L. infantum* in males, and evidence suggests this could be attributed to a higher mortality of infected females, in which pregnancy and the nursing period are relevant factors to be considered [59]. Moreover, oestrus has been associated with a diminished immune response [60, 61].

The main risk factor associated with *L. infantum* infection is the presence of sand flies, which is directly related to geographical area, weather variables, habitat, the presence of other reservoirs and whether preventive measures are used against the vector [11, 12]. Here, it was observed that seropositivity was notably higher in dogs living outdoors, the risk of testing positive being 2.86 times higher and related to greater exposure to the vector. This, along with living in an endemic area could increase the likelihood of infection [12, 58]. However, other studies have not found differences related to habitat [43, 57]. When the use of ectoparasiticides was considered, *L. infantum* seroprevalence was significantly higher in animals not subjected to prophylactic measures, confirming the repellent and/or insecticidal efficacy of pyrethroids against sandflies [11, 52, 62].

### *Dirofilaria immitis* infection

Overall positivity toward *D. immitis* in this study was 3.2%, with regional variation ranging from 0% to 24.4%. This wide prevalence range is determined by climate factors, such as high temperatures and humidity, especially in irrigated crop areas providing moist habitats which are favourable for mosquito breeding [16, 63]. Although previous predictive models have identified high-risk areas across Spain, including parts of the southeast and island territories [63], our findings indicated a lower prevalence in southeastern regions (Murcia and parts of Andalusia), and the Canary Islands. This decline may be attributed to the implementation of preventive measures and pet owner awareness [10, 19, 29]. In contrast, higher rates in northern regions, such as Soria and Navarra, may reflect limited prevention efforts [29, 44] and recent environmental changes, including increased irrigation [16, 63].

Heartworm disease is chronic in dogs, and its clinical signs develop gradually. Initially, the disease is often subclinical, but over time, it progresses to present clinical signs, such as moderate-to-severe dyspnoea, weakness, exercise intolerance, and syncope. In this study, 64% of positive dogs were clinically healthy, while only 9% exhibited severe clinical signs, suggesting that many infected dogs can pass unnoticed, contributing to ongoing transmission in endemic areas. Our finding consistent with studies from Portugal, where 4% of apparently healthy dogs were infected [64]. These findings emphasize the need for routine diagnostic testing of dogs living in or travelling to endemic areas of dirofilariosis, as clinical signs may not always be apparent. Moreover, infected dogs may not show signs until the disease has reached an advanced stage. In endemic areas, early infection detection through annual testing is crucial to prevent severe complications, such as heart failure and pulmonary hypertension [22],once again highlighting the potential role of apparently healthy dogs in the transmission and persistence of heartworm disease.

We here found that prevalence was significantly associated with age, being lower in dogs younger than 1 year. This was expected as the estimated prepatent period is 120–180 days [65]. No significant differences were detected in relation to sex. In contrast, a higher prevalence was observed in non-neutered dogs, as observed with *L. infantum* seropositivity rates. Additionally, indoor dogs showed a lower infection risk likely due to reduced exposure to the mosquito vector. However, it must be taken into account that other prophylactic measures are necessary, such as the use of window and door nets and repellents, since, as confirmed by Montoya-Alonso et al., cats in the Barcelona metropolitan area without outdoor access showed an 11.5% prevalence of *D. immitis* infection [66]. Preventive measures (macrocyclic lactones and ectoparasiticides) have proved effective in controlling this infection in endemic areas, such as Canary Islands, where its prevalence has been significantly reduced in the past decades from up to 60% to less than 20% [10, 16, 18, 29, 67]. Similar data were obtained in this study, in which the prevalence of *D. immitis* infection was significantly higher in dogs not subjected to prophylactic measures as described previously [36, 68].

### *Ehrlichia canis* seropositivity

The seroprevalence of *E. canis* observed in this study was slightly lower than that recorded in previous CVBD surveys in Spain [28, 29], yet comparable to the 3.1% prevalence found by Miró et al. (2022) in a large-scale European study [56]. The overall distribution pattern, however, was similar and significantly higher in southern and eastern areas, where *Rhipicephalus sanguineus,* the main vector, is well-established [69–71]. This vector’s distribution is driven by environmental factors, such as temperature and humidity, which affect the epidemiology of canine monocytic ehrlichiosis.

Canine monocytic ehrlichiosis (CME) typically progresses through acute, subclinical and chronic phases. The subclinical stage may persist for months or even years; during this phase, dogs generally show no clinical signs other than thrombocytopenia, making diagnosis difficult [37, 72, 73], particularly in endemic areas, where many dogs remain subclinically infected [32, 74]. Our findings are in line with these observations in that a significant proportion of seropositive dogs (35.9%) were clinically healthy, reinforcing the importance of routine screening.

Regarding epidemiological factors, according to prior results, CME may appear at any age and affect both sexes [32]. However, some studies have detected a higher seroprevalence in young dogs (under 1 year) [28], while others have noted higher seropositivity rates in older animals [29, 36]. The latter may be attributed to a higher probability of exposure to *E. canis* as a dog ages, rather than to an increase in susceptibility with age [32]. No significant sex predisposition was detected to *E. canis* infection, although a higher seropositivity was observed in non-sterilized dogs, attributable to greater exposure to vectors due to behavioural characteristics [75].

As for the other two vector-borne pathogens, the seroprevalence of *E. canis* infection was found higher in animals housed outdoors, as they are exposed for a longer period to the vector. In addition, it was noted here that seroprevalence was higher in those animals that had ticks when clinically examined by the veterinarian. Furthermore, an effect was observed of the antiparasitic drugs used to control ticks in dogs, as dogs treated with tick repellents and acaricides showed a lower prevalence of infection. In agreement with global studies, *E. canis* prevalence is higher in dogs not undergoing preventative measures against ticks. In Russia, a study found a low seroprevalence (0.2%), with no positive cases recorded among dogs receiving prophylactic treatment [24]. Similarly, in another study conducted in Italy, a significantly higher seroprevalence was observed in dogs living in public shelters than private ones, where they were regularly treated against tick infestations [25, 62]. These findings highlight the need for consistent tick control strategies to mitigate CME transmission.

Co-infections are common with CVBDs and are associated with the presence of competent vectors, a lack of protection against ectoparasites, and a longer exposure time (e.g., more time spent outdoors). In this study, 1.3% of dogs tested positive for more than one pathogen. These co-infections may significantly influence disease presentation, severity, and response to treatment, thereby complicating diagnosis and clinical management. The results of the present survey revealed a higher prevalence of clinical CanL and CME as observed previously [29, 76, 77]. Hence, if a CVBD is diagnosed by the veterinarian, other endemic CVBDs in the geographic area, where the dog is living or has travelled should also be assessed, because, as seen in the present study, clinical signs are not always observed in seropositive animals. Our data support the hypothesis that simultaneous positivity to vector-borne pathogens contributes to more severe or atypical clinical signs. These statistically significant findings confirm that co-positivity increases the likelihood of exhibiting compatible clinical signs, emphasizing the synergistic pathogenic effects of these vector-borne agents. Specifically, the proportion of dogs with compatible clinical signs was significantly higher in dogs seropositive to *L. infantum* and *E. canis*, as well as among those positive to *L. infantum* and *D. immitis*. Consequently, the clinical picture and severity in dogs positive for more than one pathogen may not be attributable to a single one.

Study limitations: This study has several limitations, primary related to the diagnostic approach based on a rapid immunochromatographic test (URANOVet®), which although suitable for large-scale field studies, may present cross-reactivity and reduced sensitivity in subclinically infected dogs, potentially leading to under or overestimation of the true prevalence. Furthermore, additional or confirmatory diagnostics such as PCR or systematic fecal examinations to rule out other nematode infections were not performed due to economic constraints. Furthermore, seropositivity for *L. infantum* and *E. canis* reflects exposure or infection rather than active clinical disease, and clinical signs cannot be unequivocally attributed to a single pathogen in the absence of systematic screening for comorbidities or co-infections. Regarding prophylactic measures, although we collected information on whether dogs received ectoparasiticides, we could not verify the specific products used, the duration of protection or owner compliance for each dog throughout its lifetime. This is a significant limitation, because positive antibody tests for *L. infantum* and *E. canis* may reflect exposure that occurred before prophylaxis was initiated. Therefore, the observed associations between prophylaxis and seropositivity should be interpreted with caution. Longitudinal studies with verified treatment records would be needed to establish causal relationships between specific preventive protocols and infection risk. Additionally, the classification of clinical signs was based on individual veterinarians' assessments rather than standardized criteria. Although we attempted to grade severity, we acknowledge the potential for inter-observer bias. Therefore, future studies should consider implementing standardized clinical scoring systems to enable more detailed comparisons. Finally, although the sampling strategy aimed for proportional geographic representation, variability in clinic participation and the possibility of selection bias cannot be fully excluded, the large nationwide sample size strengthens the robustness of the overall findings.

## Conclusions

The results of the present study indicate that *Leishmania infantum*, *Dirofilaria immitis* and *Ehrlichia canis* remain relevant vector-borne pathogen infections among dogs in Spain, with notable geographical variation in their prevalence. This variation is in line with known patterns of vector distributions and is likely influenced by climate diversity and thus bioclimatic zones. Our results highlight the importance of region-specific diagnostic protocols and control strategies. Identifying subclinical infections enables timely intervention, mitigates disease progression, improves treatment outcome, and ultimately contributes to the broader goal of controlling vector-borne diseases in both animals and humans. As a key step in the differential diagnosis of these diseases, we would also recommend considering the detailed travel history and place of origin of all dogs, especially those living in or travelling to endemic areas.

## Supplementary Information

Supplementary material 1.

Supplementary material 2.

## Data Availability

Data supporting the main conclusions of this study are included in the manuscript.

## Abbreviations

CanL: Canine leishmaniosis
CI: Confidence interval
CME: Canine monocytic ehrlichiosis
CVBD: Canine vector-borne disease
Di: *Dirofilaria immitis*
Ec: *Ehrlichia canis*
Li: *Leishmania infantum*
OR: Odds ratio
