# Supplementary material for: Nationwide seroprevalence of canine vector-borne pathogens in dogs in Spain
Source: Parasit Vectors. 2026 Jun 14;19:344. doi: 10.1186/s13071-026-07500-3 (PMC13491873; doi:10.1186/s13071-026-07500-3)
Supplement: Supplementary file 1 [file 13071_2026_7500_MOESM2_ESM.pdf]

FORMULARIO DE RESULTADOS ESTUDIO SOBRE LA PREVALENCIA DE LA INFECCIÓN POR *LEISHMANIA INFANTUM, EHRLICHIA CANIS Y DIROFILARIA IMMITIS*

Nombre persona contacto:

Clínica Veterinaria:

Dirección:

Población/Provincia/CP:

Teléfono de contacto:

Correo electrónico

Para participar en el estudio:

1. Utilizar Test en todos los perros compatibles con infección (animales enfermos, anemia, trombocitopenia, fiebre, malestar, cojeras, ...) o en perros clínicamente sanos como screening.
2. Rellenar los resultados en la tabla adjunta.
3. Enviar este formulario de resultados cumplimentado, escaneado por correo electrónico a Ana Montoya amontoya@ucm. Guardar el impreso original para posterior recogida por el delegado de Bayer o envíoa por correo postal franqueo pagado (sobres disponibles a través del delegado de Bayer).

**Todos los resultados deberán proceder del análisis realizado con URANO TEST

Observaciones: anote todos los datos de interés asociados a este caso clínico que considere en el reverso de esta ficha clínica.
Si es posible, identificar y guardar en refrigeración el tubo con la muestra restante así como las garrapatas presentes en el perro para posibllitar posterior recogida.

						Resultado del Test			Signos clínicos (0-3)			Presencia de ectoparásitos		Uso de antiparasitario externo	
Muestra	Nombre del Paciente/ Apellido del propietario	Fecha de extracción (día/mes)	Edad en Años	Sexo	Habitat (exterior ó interior)	*L. infantum*	*D. immitis*	*E. canis*	*L. infantum*	*D. immitis*	*E. canis*	Sí/No	Especificar (Pulga/Garrapata)	Sí/No	Especificar
0	Toby / Martínez	02-jun	5	MC	EXT	POS	POS	NEG	3	0	1	Sí	Pulga	Sí	Advantix
1															
2															
3															
4															
5															
6															
7															
8															
9															
10															
11															
12															
13															
14															
15															

☐ **Acepto el uso de mis datos para la elaboración del informe final**

El formulario de resultados no será válido sin los datos, el sello y la firma

Sexo:
M = Macho
MC = Macho castrado
H = Hembra
HO = Hembra esterilizada

Signos clínicos
0= sano
1= leve
2= moderado
3= grave

Nombre y firma sello:_______________________________

CARTA DE ACEPTACIÓN DE PARTICIPACIÓN EN EL ESTUDIO EPIDEMIOLÓGICO PROMOVIDO POR BAYER HISPANIA, S.L. TITULADO “PREVALENCIA DE *LEISHMANIA INFANTUM, EHRLICHIA CANIS Y DIROFILARIA IMMITIS* EN ESPAÑA”

En ………….., a … de …………………. de 2016

Nombre y Apellidos: ____________________

N.I.F.: ____________________

Dirección: ____________________

Dirección de E-mail: ____________________

Teléfono de contacto: ____________________

1. Manifiesto que conozco el estudio arriba referenciado cuyo objetivo principal es conocer la prevalencia de ehrlichia, leihsmania, dirofilaria en España y que he recibido de Bayer Hispania, S.L. (Bayer) la siguiente documentación y material para llevar a cabo el Estudio:

   - Protocolo del estudio titulado: ***“Prevalencia de leishmania infantum, ehrlichia canis y dirofilaria immitis en España”*** (en adelante el Estudio)
   - Formulario de Recogida de Datos.
   - Kits de diagnóstico Urano para la detección de leishmania infantum, *ehrlichia canis y/o dirofilaria immitis* necesarios para poder llevar a cabo el Estudio.

2. Declaro que cuento con la suficiente experiencia y capacitación para llevar a cabo el Estudio y que mi participación en el mismo no constituye un incentivo para la recomendación, prescripción, compra, suministro, venta o administración de un determinado medicamento.

3. Me comprometo a llevar a cabo mi participación en el Estudio cumpliendo en todo momento con la normativa vigente aplicable así como con lo dispuesto en las normas de sanidad animal, éticas y de Buena Práctica aplicables. Asimismo, me comprometo a no utilizar con fines comerciales el material del Estudio que Bayer ha puesto a mi disposición.

4. Manifiesto que conozco y acepto que la propiedad de los resultados del Estudio corresponden a Bayer cuyos derechos de propiedad industrial y/o intelectual se reserva.

5. Me declaro informado de que mis datos personales serán incorporados a un fichero de propiedad de Bayer cuyas finalidades son las necesarias para la correcta gestión del Estudio y que podré ejercitar mis derechos de acceso, rectificación, cancelación y oposición mediante Internet a la dirección Oficinaprotecciondatos@bayer.es o por correo mediante escrito dirigido a Oficina de Protección de Datos, BAYER HISPANIA, S.L. Avd. Baix Llobregat 3-5, C.P. 08970 Sant Joan Despí, Barcelona.

6. Acepto los términos en que se va a desarrollar el Estudio y que están establecidos en el Protocolo y en la demás documentación arriba descrita, firmando el presente documento en duplicado ejemplar y a un solo efecto, en señal de conformidad expresa con todos los términos del mismo, en el lugar y fecha arriba indicados.

Fdo.:

……………………………………………..
